# A Recombinant *Shigella flexneri* Strain Expressing ETEC Heat-Labile Enterotoxin B Subunit Shows Promise for Vaccine Development via OMVs

**DOI:** 10.3390/ijms252312535

**Published:** 2024-11-22

**Authors:** Josune Salvador-Erro, Yadira Pastor, Carlos Gamazo

**Affiliations:** Department of Microbiology and Parasitology, Navarra Medical Research Institute (IdiSNA), University of Navarra, 31008 Pamplona, Spain; jsalvador.1@alumni.unav.es (J.S.-E.); ypastor@alumni.unav.es (Y.P.)

**Keywords:** *Shigella flexneri*, enterotoxigenic *Escherichia coli* (ETEC), outer membrane vesicles (OMVs), recombinant vaccine, heat-labile enterotoxin B (LTB)

## Abstract

Diarrheal diseases caused by *Shigella* and enterotoxigenic *Escherichia coli* (ETEC) are significant health burdens, especially in resource-limited regions with high child mortality. In response to the lack of licensed vaccines and rising antibiotic resistance for these pathogens, this study developed a recombinant *Shigella flexneri* strain with the novel incorporation of the *eltb* gene for the heat-labile enterotoxin B (LTB) subunit of ETEC directly into *Shigella*’s genome, enhancing stability and consistent production. This approach combines the immunogenic potential of LTB with the antigen delivery properties of *S. flexneri* outer membrane vesicles (OMVs), aiming to provide cross-protection against both bacterial pathogens in a stable, non-replicating vaccine platform. We confirmed successful expression through GM1-capture ELISA, achieving levels comparable to ETEC. Additionally, proteomic analysis verified that the isolated vesicles from the recombinant strains contain the LTB protein and the main outer membrane proteins and virulence factors from *Shigella*, including OmpA, OmpC, IcsA, SepA, and Ipa proteins, and increased expression of Slp and OmpX. Thus, our newly designed *S. flexneri* OMVs, engineered to carry ETEC’s LTB toxin, represent a promising strategy to be considered as a subunit vaccine candidate against *S. flexneri* and ETEC.

## 1. Introduction

Diarrheal infectious diseases constitute a serious global health problem. They are one of the leading causes of death in children under five years old, responsible for 443,832 deaths annually in this age group [1]. These diseases have a particularly high mortality rate in low-resource countries, primarily due to limited healthcare infrastructure and restricted access to treatments such as antibiotics and preventive measures like vaccines. Mortality from these diarrheal diseases was traditionally associated with severe dehydration and fluid loss, but the current disease paradigm has shifted, with the ability for bacterial infections to cause sepsis now posing a greater risk of death [1].

Among the main etiological agents responsible for diarrhea, *Shigella* spp. and enterotoxigenic *Escherichia coli* (ETEC) account for 10.8% and 3.9% of cases, respectively [2], being the most impactful bacterial species in diarrheal diseases. Due to the high burden of these pathogens, the development of vaccines against them is a priority, as highlighted by the WHO’s Product Development for Vaccines Advisory Committee (PDVAC) and Immunization, Vaccines, and Biologicals Advisory Committee. This commission has recently developed guidelines on Preferred Product Characteristics, assessing the key characteristics that vaccines must possess for both pathogens individually, with the common objectives of safety, efficacy, and economic accessibility [3]. The urgency of vaccine development is amplified for two reasons: first, there are currently no vaccines on the market against these bacteria, and second, the drastic increase in antibiotic resistance, which is especially problematic in the case of *Shigella* spp. and *E. coli* spp., listed among the top ten bacterial species in the WHO Bacterial Priority Pathogens List of 2024 [4].

ETEC is a key cause of traveler’s diarrhea [5]. After ingestion and reaching the small intestine, ETEC releases different enterotoxins responsible for the onset of diarrhea, such as the heat-labile toxin (LT) and the heat-stable toxin (ST) which can be produced both simultaneously and individually, each exhibiting diarrheagenic activity independently [6].

The ST is a low-MW cysteine-rich peptide. This enterotoxin has proven to be poorly immunogenic, thus limiting its potential as a vaccine candidate. Even though recent efforts have begun to reintroduce it with certain genetic mutations, few vaccine candidates currently include this toxin [6].

On the other hand, LT has a higher MW of 84 kDa, encoded by the *eltab* operon. It consists of a heterohexameric AB5-type molecule, with great structural and functional similarity to cholera toxin (CT), sharing 80% homology [7]. It is formed by two subunits: the A subunit (LTA), which exhibits catalytic activity and is the active part of the toxin, and the pentameric B subunit (LTB), responsible for binding to the monosialoganglioside GM1 receptor on the surface of the enterocyte [8]. LTB has been extensively studied due to its immunogenic capacity, producing specific anti-LT antibodies and stimulating cytokine production. This, combined with its lack of toxicity and adjuvant capacity as immunomodulator, makes it an excellent vaccine candidate [7,9,10].

In contrast, the genus *Shigella* is the etiological agent of human shigellosis, also known as bacillary dysentery. This genus is closely related to *E. coli* and is classified into different species, with *S. flexneri* being endemic in developing countries [11]. It is an intracellular pathogen that infects epithelial cells of the colon, replicates within them, and then spreads to adjacent cells [12]. This process triggers cellular secretion of IL−8, followed by neutrophil recruitment, leading to inflammation, destruction of the epithelium, and the characteristic blood- and mucus-containing diarrhea [13].

Like most Gram-negative bacteria, *Shigella* releases outer membrane vesicles (OMVs) into the environment. These vesicles contain different virulence factors, including outer membrane proteins (Omps), adhesins, Ipa proteins, and lipopolysaccharide, which are involved in the invasion process [14], but also are well-recognized “pathogen associated molecular patterns”, which makes OMVs excellent vaccine candidates, as demonstrated in previous studies where protection was achieved in murine models via different administration routes [14,15,16].

Despite the developing efforts and the progress of some vaccine candidates to phase 1 trials, there is currently no licensed vaccine against these two bacterial species [17]. Due to the lack of preventive tools and in response to current global needs outlined by the WHO [18,19], our goal is to design and develop a single subunit vaccine capable of protecting against both bacterial species, ETEC, and *S. flexneri*. To achieve this objective, the *S. flexneri* and *S. flexneri ΔtolR* strains were employed for cloning, the latter being an attenuated recombinant strain developed by our research group that, due to its mutation in the Tol-Pal system, has an enhanced capacity to release vesicles, which is of interest in the production of our antigenic complex [16]. This vaccine candidate aims to incorporate main antigens from each bacterium, specifically the LTB subunit toxin from ETEC and *S. flexneri* vesicles. Here we describe the development of a recombinant *S. flexneri* strain engineered to express LTB, facilitating the co-delivery of *Shigella* and ETEC antigens through *S. flexneri* vesicles. The potential benefits of vesicles include enhanced safety due to their non-replicating nature and improved stability, making them easier to store and transport compared to traditional vaccines.

## 2. Results

### 2.1. Detection of ETEC LTB Protein in Recombinant Shigella flexneri Strains

**Figure 1:**
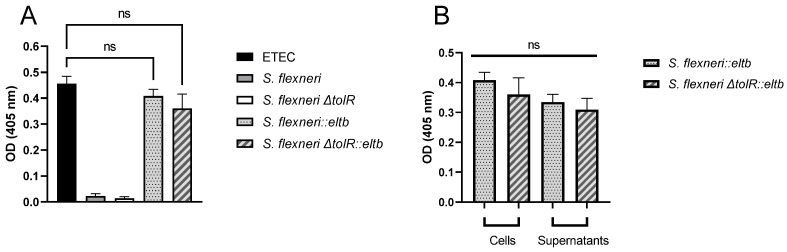
LTB detection in ETEC and different parental or transgenic *Shigella flexneri* strains using a GM1 capture-ELISA. (**A**) The presence of LTB was confirmed on ETEC and *S. flexneri::eltb* and *ΔtolR::eltb* strains. (**B**) In LTB-positive samples, no significant differences were observed between cell-associated and bacterial culture supernatant fractions. [ns, not significant (*p* ≥ 0.05)].

Recombinant construction of *S. flexneri* strains expressing ETEC LTB subunit protein was achieved by incorporating the *eltb* promoter region and gene from ETEC into *S. flexneri* wild-type (wt) and *ΔtolR* strains, as described above. To confirm the successful insertion of the *eltb* gene and the resulting constitutive protein expression, a specific GM1-capture ELISA was conducted. As shown in Figure 1A, the protein was detected in bacterial cultures on both genetically modified *Shigella* strains, *S. flexneri::eltb* and *S. flexneri ΔtolR::eltb,* as well as on the ETEC control strain. Strikingly, these transgenic strains showed elevated constitutive expression levels of LTB similar to ETEC, with no significant differences observed between them. Protein presence in these samples was analyzed following bacterial inactivation with BEI/FA, while no signal was detected after heat inactivation. As expected, no signal was detected when parental *S. flexneri* and *S. flexneri ΔtolR* were used (Figure 1A).

To more deeply characterize which fraction of the bacterial culture contained the LTB protein, both bacterial cells and culture supernatants were analyzed. LTB protein distribution was relatively homogeneous across both fractions as no significant differences were observed between samples (Figure 1B).

### 2.2. Outer Membrane Vesicles Characterization

#### 2.2.1. Size Distribution and Zeta Potential

From each parental and transgenic strain, OMVs were collected after bacterial inactivation using BEI-FA, and HT-OMVs were obtained after heat treatment of the bacterial cultures (see above). A Malvern Zetasizer was used to determine the size distribution and zeta potential of the different vesicle products. Mean size distribution and Z potential of the outer membrane vesicles derived from parental and LTB-recombinant strains are indicated in Table 1. The analysis revealed vesicles with diameters of roughly 200 nm and a more electronegative potential of the vesicles obtained from the recombinant strains.

**Figure 2:**
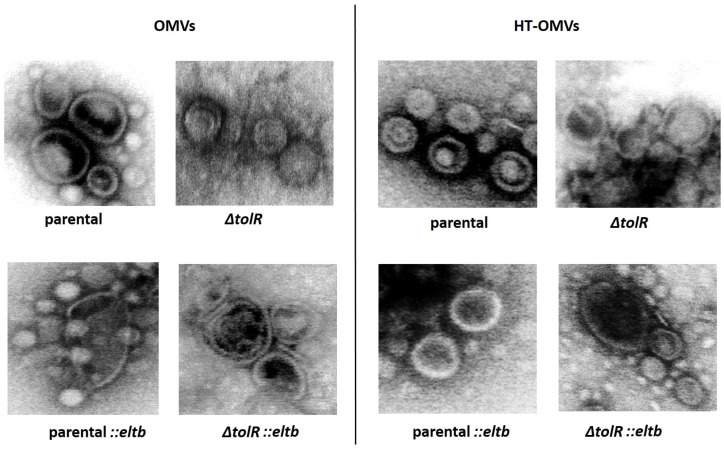
Representative transmission electron microscopy (TEM) images of OMVs and HT-OMVs isolated from parental *Shigella flexneri*, *S. flexneri ΔtolR* mutant, and their respective recombinant containing *eltb* (scale bar = 200 nm).

Using transmission electron microscopy, we identified spherical structures within the anticipated size range of OMVs and HT-OMV (Table 1). Figure 2 displays representative images from the parental and recombinant vesicles containing LTB protein.

#### 2.2.2. Proteomics

**Figure 3:**
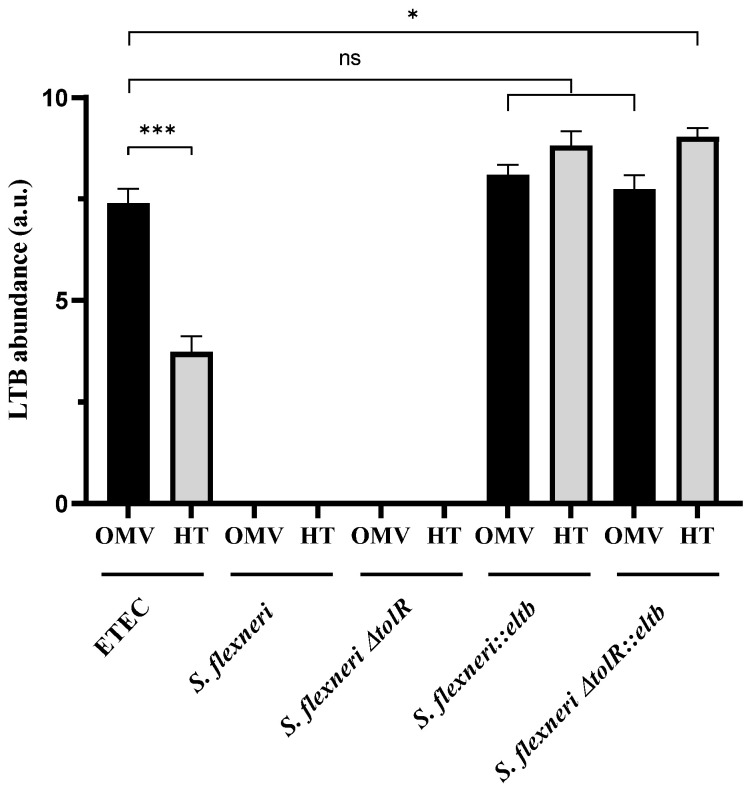
Proteomic analysis identified LTB abundance in the different OMV and HT products from ETEC and *Shigella flexneri* or mutant (*ΔtolR*), with and without the *eltb* insertion. Samples were analyzed in triplicates and the protein abundance was normalized to the total protein (*, *p* < 0.05; ***, *p* < 0.001; ns, non-significant; a.u.: arbitrary units).

Proteomics analysis confirmed the presence of the LTB protein (Figure 3). As expected, the analysis confirmed the expression of the protein in both ETEC native products and in *Shigella::eltb*-mutated samples, but not in *Shigella*-derived vesicles. OMVs from ETEC obtained by chemical inactivation expressed higher levels of LTB (*p* < 0.001) compared to HT-OMVs vesicles, although this difference was not observed in *Shigella* OMV/HT.

**Figure 4:**
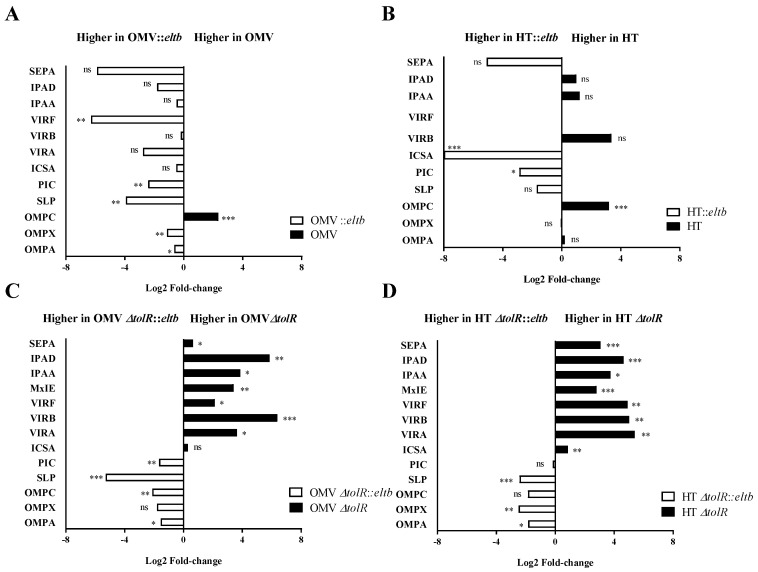
Differential expression of the main outer membrane proteins and virulence factors included in OMV and HT products from *Shigella flexneri* wild-type (**A**,**B**) or mutant *ΔtolR* (**C**,**D**) before (black) and after *eltb* mutation (white). Proteins were clustered in two groups based on their expression profile. Samples were analyzed in triplicates and the Log2 of the fold change is represented (*, *p* < 0.05, **, *p* < 0.01, ***, *p* < 0.001; ns, non-significant).

A comparative proteomic analysis was also conducted to identify significant alterations in the overall proteome profile of the recombinant strains. The quantitative analysis confirmed that the main outer membrane proteins and virulence factors from *Shigella* are present in all the samples tested in the study, including OmpA, OmpC, IcsA, SepA, and Ipa proteins, among others. However, some differences in protein expression were observed. When comparing OMVs and HT-OMVs from the wild-type with the corresponding from the *Shigella::eltb* strains, the latter was enriched in some Omps such as Slp, OmpA, and OmpX, and in some virulence factors like VirF and IcsA, although Ipa protein’s expression was non-significant. Only OmpC appeared to be overexpressed in wild-type and HT-OMVs vesicles (*p* < 0.001) (Figure 4).

Conversely, regarding the *Shigella ΔtolR*-derived vesicles, the protein profile changed remarkably with and without the *eltb* mutation. As shown in Figure 3, both OMVs and HT-OMVs, including LTB, are enriched (*p* < 0.05) in Omps, whereas the expression of virulence factors was significantly decreased in these vesicles compared to *Shigella ΔtolR* derived vesicles.

### 2.3. Bacterial Growth Analysis and Antibiotic Sensitivity

**Figure 5:**
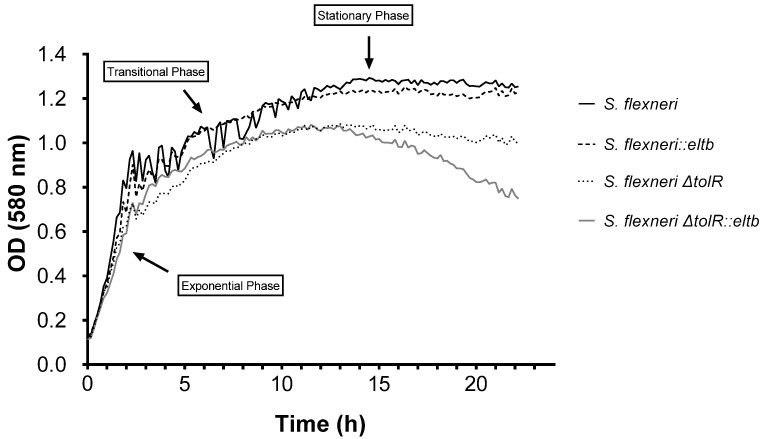
Bacterial growth curves of *Shigella flexneri* wt, *S. flexneri::eltb, S. flexneri ΔtolR, and S. flexneri ΔtolR::eltb.* Bacterial suspensions were inoculated on Bioscreen C multi-well plates and incubated with continuous shaking at 37 °C. Absorbance values (O.D._580 nm_) were automatically read at regular intervals of 10 min for 22 h. An arbitrary position of the stationary phase is indicated according to the growth curve kinetics.

The phenotypic analysis of the recombinant strains was extended by examining growth curves in vitro and assessing antibiotic sensitivity to investigate potential pleiotropic effects of the *eltb* insertion gene. The analysis showed similar growth kinetics of the recombinant strains compared to their parental strains (Figure 5), with similar generation time (30 ± 1 min). However, *ΔtolR* mutant strains reached the stationary phase with lower absorbance levels compared to *S. flexneri* wt and *S. flexneri::eltb* strains. Additionally, the double recombinant strain exhibited a shorter stationary phase, entering the death phase at 13 h, whereas this phase was not observed in the other strains within the 22 h experiment.

An antibiotic sensitivity study was also performed. First-line antibiotics for shigellosis, such as ciprofloxacin and ceftriaxone, as well as fosfomycin, ertapenem, and aztreonam, among others, were tested using the Vitek 2 system (bioMérieux, Madrid, Spain). No significant differences were obtained between the recombinant and the wild-type strains in any of the antibiotics tested in the study, presenting identical minimum inhibitory concentrations (MICs).

### 2.4. Biofilm Formation Capacity

**Figure 6:**
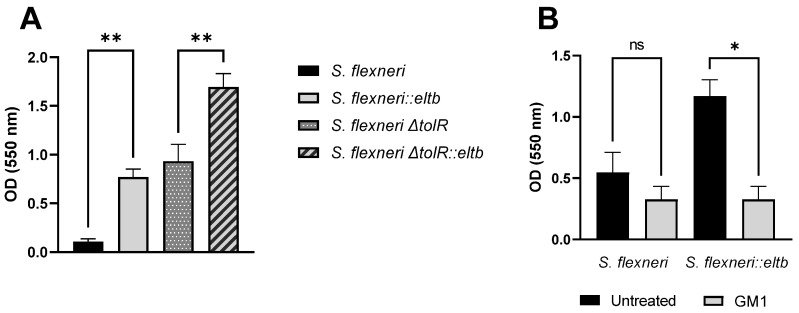
Biofilm formation assay results. (**A**) Comparison of biofilm formation among recombinant and parental *Shigella flexneri* strains, showing significant increases in biofilm production for the *S. flexneri::eltb* and *S. flexneri ΔtolR::eltb* strains (**, *p* < 0.01) compared to the parental strains. (**B**) Evaluation of LTB involvement in biofilm formation, showing a significant decrease in biofilm formation capacity after the addition of GM1, the natural receptor of LTB (*, *p* < 0.05), with no significant differences observed in the parental strain (ns).

Considering the surface changes in the vesicles described previously, we were interested in studying whether the corresponding bacteria would have an altered ability to form biofilms. Taking the parental strains as a reference, we conducted a biofilm formation assay using the novel *Shigella::eltb* strains obtained in this study. Figure 6 shows that *Shigella* strains that had incorporated the *eltb* gene exhibited significantly higher biofilm formation rates (*p* < 0.01) compared to their parental strains. To demonstrate the implications of the LTB protein, another assay was conducted using its natural receptor, GM1, which we utilized as a blocker. The results showed a reduction in the amount of biofilm formed upon the addition of GM1 to the medium in the strains expressing this protein, whereas no effect was observed in the control strains.

### 2.5. Cell Viability Assay

**Figure 7:**
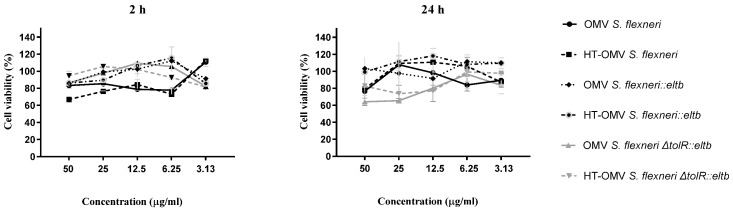
Effect of OMVs and HT-OMVs on the mitochondrial activity (MTT assay) on HeLa cells. Figures indicate the percentage (%) of mitochondrial activity after treatment for 2 or 24 h with different concentrations of OMVs and HT-OMVs (μg/mL) compared to untreated cells. Experiment was performed in triplicate. Error bars represent SEM.

In order to evaluate the cytotoxicity of the vesicles, HeLa cells were treated with different concentrations (ranging from 3 to 50 μg/mL) of OMV or HT-OMV products derived from the different *Shigella* strains. Results showed no significant differences between samples at 2 or 24 h of incubation. The cell viability was above 60% even at the highest concentrations, increasing up to 90–100% as concentrations decreased (Figure 7).

## 3. Discussion

A combined vaccine against *Shigella* and ETEC is a priority strategy to face the leading causes of diarrhea worldwide. Although the WHO has recently reaffirmed this strategy and set the Preferred Product Characteristics (PPCs) for the vaccine [18,19], little effort has been invested into developing a safe, single, multiepitope candidate [20]. To our knowledge, only two oral live attenuated candidates have been developed and progressed to Phase 1 clinical trials: CVD 1208S-122, consisting of an attenuated *S. flexneri 2a* strain expressing the ETEC colonization factor antigen-1 (CFA/1) and the LTB [21], and ShigETEC, an oral live attenuated *Shigella* that contains the toxins LT and ST of ETEC [22]. Since the LTA subunit is toxic, the B subunit of the enterotoxin is considered as the main component in ETEC vaccination, demonstrating good safety and high immunogenicity in clinical trials [20].

In previous studies, we described novel OMV or H-OMVT products, obtained both from wt or isogenic mutant *S. flexneri ∆tolR* strains, with increasing capacity to release OMVs, which contain the main antigens of *Shigella* and are able to generate protective antibody-mediated and cellular immune responses in mice [16,23]. Based on these previous studies and with the aim of obtaining a multi-pathogen subunit vaccine against *Shigella* and ETEC, here we engineered two novel *S. flexneri* strains inserting the *eltb* gene from ETEC in the chromosome of *Shigella* as OMV-producing platforms. Thus, we present different OMV products containing the main *Shigella* immunodominant proteins and the LTB subunit of LT enterotoxin. To our knowledge, only one single study has developed a *Shigella* strain containing LTB, but the protein was cloned on the large invasion plasmid of *S. flexneri* to obtain an attenuated vaccine [22]. It has been described that *Shigella* invasion plasmid is unstable and can be lost partially or even completely during in vitro culture or environmental conditions [24], increasing the risk of losing the exogenous genes. For that reason, an insertion which induces a constitutive expression of the LTB enterotoxin is desired.

Here, the *eltb* insertion and following expression in both wt and *∆tolR* mutant *Shigella* genomes was corroborated by GM1-ELISA, obtaining similar LTB expression between the ETEC control strain and *Shigella::eltb* mutants. Furthermore, in this assay, we demonstrated that LTB was detected in both bacterial the fraction and supernatant of the cells with no significant differences, confirming that *Shigella* is able to release the acquired ETEC protein from its cytosol. In ETEC, LTB has been described to be released by the Type II Secretion System (T2SS) [25]; however, to date, this effector delivery system has only been described in *S. boydii* and *S. dysenteriae* but not in *S. flexneri* [26], so the mechanism by which this toxin is released requires further investigation.

Also, here we present two different procedures to obtain OMVs based on our previous research: vesicles recovered from the culture medium after a chemical bacterial inactivation process (from which we obtain native OMVs) or after a heat treatment (from which we obtain HT-OMV product) both obtained from a wt or a *∆tolR* mutant *S. flexneri.* Size analysis of all the antigenic complexes corroborated that HT products are smaller than native OMVs [15], and that *∆tolR* derived products are bigger than the ones from wt strains [16]. Here, these results are consistent, with no significant differences compared to the new *eltb*-containing vesicles, demonstrating that this insertion does not affect the size of the OMVs.

Once the presence and production of LTB by wt and *∆tolR Shigella* strains was demonstrated, we further investigated if the protein was present in vesicles. Previous proteomic studies of the group already demonstrated that LTB could be found in the vesicles of ETEC [27]. Accordingly, here we confirmed that OMVs and HT-OMVs produced by *Shigella* mutant strains also contained the LTB protein (Figure 3).

Apart from the LTB presence, proteomic analysis of the different vesicle products confirmed the conservation of the main outer membrane proteins of *Shigella*, such as OmpA, OmpC, Slp, and IcsA, and other important virulence factors, including Ipa and Vir proteins, involved in cell adhesion, invasion, and dissemination of the pathogen [28]. Interestingly, the study revealed that the insertion of the *eltb* gene in *Shigella* genome did affect the expression levels of many of these proteins in the ∆*tolR* mutant strains. Thus, the OMV/HTs derived from *∆tolR::eltb Shigella* strains presented significantly higher levels of OmpA, with known immunomodulation properties and a main antigen component in *Shigella* vaccine candidates [29].

Taking this together, the results indicate that *eltb* insertion may have an effect on the protein content and membrane composition of *S. flexneri.* Accordingly, the effect of this enterotoxin subunit on bacterial survival and virulence was evaluated. First, we observed that the insertion did not affect bacterial viability and growth, showing the same generation time compared to wt or mutant strains. Second, and based on the higher capacity of ETEC to form biofilms compared to *Shigella* strains [30], we hypothesized that the acquisition of the *eltb* gene could alter this property on the recombinant strains. Interestingly, results showed that *eltb* recombinant strains showed significantly higher (*p* < 0.01) biofilm formation capacity compared to their parental strain (Figure 6A). LT has been shown to facilitate ETEC pathogenesis by promoting initial adherence and colonization of the intestinal mucosa [31,32]. It can bind simultaneously to both bacterial and host cell surfaces, via LPS molecules on the bacterial side, and the GM1 ganglioside on the host side, since its interaction with LPS occurs in a domain distinct from the GM1-binding site [33,34]. Although some studies suggest that the expression of LT holotoxin significantly enhances adherence compared to the LTB subunit alone [35], our findings indicate that LTB alone is sufficient to induce significant bacterial aggregation and adherence in vitro (Figure 6B), effectively promoting biofilm formation, without causing toxicity.

However, since *∆tolR* mutant also presented higher capacity of biofilm formation than wt, other factors may also contribute to this function, such as the different protein expression of the bacterial membrane, which is in accordance with the higher presence of some proteins that promote the biofilm formation, such as IcsA, which are increased in the mutant strains [36].

In this framework, LTB subunits have been widely used as adjuvants with various antigens, enhancing antigen uptake and presentation to elicit a strong immune response that boosts both humoral and cell-mediated immunity [37]. This versatility makes LTB particularly valuable for our goal, that is, the oral administration, as it promotes a strong immune response at mucosal surfaces, which are the primary defense against these enteropathogens.

Finally, in order to ensure the safety of the products and that the B subunit does not increase toxicity, in vitro cell viability assays were performed. Results demonstrated that all the samples elicited cell activation with no cytotoxic effect, confirming that the presence of LTB on the OMV antigenic complexes does not affect the viability of epithelial cells.

## 4. Materials and Methods

### 4.1. Bacterial Strains and Culture Conditions

The parental strains used in this study were Enterotoxigenic *E. coli* H10407 (ATCC 35401), *Shigella flexneri* 2a, a clinical isolate (“Clínica Universidad de Navarra”, Pamplona, Spain), and a *Shigella flexneri ΔtolR* mutant previously developed by our research group [16]. Cryobeads (Microkit laboratories, Madrid, Spain) stored at −80 °C containing the strains *S. flexneri* 2a, *S. flexneri ΔtolR*, *S. flexneri::eltb*, *S. flexneri ΔtolR::eltb*, and ETEC were incubated on Tryptone Soy Agar (TSA, Scharlab, Barcelona, Spain) and subsequently on Tryptic Soy Broth (TSB, Condalab, Madrid, Spain). Incubations were carried out at 37 °C with shaking at 140 rpm for 24 h.

### 4.2. DNA Manipulations and Mutant Construction

Genomic sequences were obtained from the Kyoto Encyclopedia of Genes and Genomes (KEGG). Primers were synthesized by Condalab (Madrid, Spain). DNA sequencing was performed by Secugen (Madrid, Spain). Restriction enzymes were used according to the manufacturer’s recommended conditions. Plasmid purification and genomic DNA extraction were carried out using the QIAprep Spin Miniprep Kit (Qiagen, Hilden, Germany) and QIAamp DNA Mini Kit (Qiagen), respectively. DNA was purified from agarose gels using the QIAquick Gel Extraction Kit (Qiagen).

To construct the recombinant LTB-producing *S. flexneri* strain, two PCR fragments were generated: *eltb*-F1 5′-TCTCCGGCATGAAAACGATG-3′ and *eltb*-R2 5′-ATGATATATAAGTTTTCCTCGAT-3′, amplifying a 176 bp fragment containing the putative promoter of the *eltab* operon located 854 bp upstream of the *eltb* gene, and *eltb*-F3 5′-ATGATATATAAGTTTTCCTCGATCAGAATTCGGAATGAATTATGAA-3′ and *eltb*-R4 5′-AGTATGGAAAACTAGTTTGC-3′, which amplifies a 399 bp fragment corresponding to the *eltb* gene present in the operon in the pLT plasmid.

After the amplification of the promoter and the *eltb* gene separately using the previously described primers, both fragments were joined via overlapping between primers *eltb*-R2 and *eltb*-F3. The resulting product was cloned into the TOPO TA cloning vector pCR2.1-TOPO (Invitrogen catalogue no. K4516-41) in Stellar cells on LB plates with X-Gal and IPTG with ampicillin (100 µg/mL to select positive mutants). This plasmid was sequenced in order to confirm the correct incorporation of the gene and subsequently subcloned using the restriction enzymes BamHI and XhoI and the suicide plasmid pUC18 R6KT mini Tn7T-Km [38]. The resulting plasmid was introduced into *S. flexneri* 2a and *S. flexneri ΔtolR* by electroporation. Screening for kanamycin resistance was used to identify transformants. Among the positive colonies, to confirm the presence of the gene, a PCR was performed with the primers *eltb*-F1 and *eltb*-R4, which amplify a 575 bp fragment in recombinant strains but do not hybridize in the parental *Shigella* strains, while in the positive control ETEC, they amplify 1327 bp. Sequencing of the different plasmids confirmed the correct isolation and cloning of the *eltb* gene. The obtaining recombinant strains *S. flexneri::eltb* and *S. flexneri ΔtolR::eltb* were stored at −80 °C in cryobeads (Microkit lab; Madrid, Spain).

### 4.3. Production and Purification of Vesicles

In order to obtain OMVs, an inoculum of 10^8^ CFU/mL of either parental or recombinant strains were incubated in 10 mL of TSB at 37 °C, 140 rpm for 24 h. Subsequently, 3 mL of the preculture was used to inoculate 3 L of TSB.

OMVs were collected after bacterial inactivation using BEI-FA (0.1 M BEA [2-Bromoethylamine hydrobromide, Sigma] formaldehyde 0.06% [Panreac, Barcelona, Spain]), as described previously [39]. Bacteria were then removed from the cultures by centrifugation at 6000× *g* for 20 min and filtration through 0.22 μm (Corning, New York, NY, USA). The supernatants containing the vesicles were concentrated by tangential ultrafiltration (polyethersulfone, 40 kDa). The retentate-containing vesicles were finally ultracentrifuged (40,000× *g*, 75 min, 4 °C) and lyophilized.

HT-OMVs (heat-treated OMVs) were obtained after heat treatment of the bacterial cultures (see above). Briefly, bacterial suspensions were steam flowed at 100 °C for 15 min. Bacteria were removed and the supernatants containing HT-OMVs were recovered as described above for OMVs. OMVs and HT-OMVs obtained were examined by transmission electron microscopy (Zeiss LIBRA 120 EFTEM, Jena, Germany).

As a result, ten extracts containing vesicles were obtained: (i) OMVs and (ii) HT-OMVs from ETEC; (iii) OMVs and (iv) HT-OMVs from *S. flexneri* 2a; (v) OMVs and (vi) HT-OMVs from *S. flexneri ΔtolR*; (vii) OMVs and (viii) HT-OMVs from *S. flexneri::eltb*; and (ix) OMVs and (x) HT-OMVs from *S. flexneri ΔtolR::eltb*.

### 4.4. Size Distribution and Zeta-Potential of Vesicles

The size, size distribution, and zeta-potential of vesicles were measured using a Malvern Zetasizer 2000 instrument (Malvern Instruments, Malvern, UK). The lyophilized samples were resuspended in deionized water, pH 7. The results were analyzed using the software ZS Xplorer (version 4.0.0). The Zetasizer measures the size distribution in a population, reflecting peaks in this distribution in nm. The experiment was performed in triplicate.

### 4.5. Growth Curves

The characteristics of the bacterial growth curve pattern of the parental and recombinant strains were obtained using Bioscreen C (Lab Systems, Lilydale, VIC, Australia) following the method previously described by Zúñiga-Ripa et al. [40]. Briefly, 200 μL/well of bacterial suspension on TSB (O.D._600 nm_ of 0.1) was inoculated on a Bioscreen C multi-well plate, with continuous shaking, at 37 °C. Absorbance values (O.D._580 nm_) were automatically read at regular intervals of 10 min for 22 h. The generation time (G) was calculated from the slope of the nonlinear regression curve (k) for each sample using the following equation:

G = ln(2)/k.

Wells containing only culture medium were used as controls.

### 4.6. Proteomic Analysis

Proteome analysis was performed by mass spectrometry to identify proteins from OMVs and HT-OMVs obtained from parental or mutant strains, from three independent batches. Briefly, samples were resuspended in a lysis buffer [8 M urea, 50 mM dithiothreitol (DTT)], diluted in Laemmli sample buffer (2% SDS, 10% glycerol, 5% 2-mercaptoethanol, 0.002% bromophenol blue, and 0.125 M Tris HCl, pH 6.8) and loaded into a 1.5 mm-thick polyacrylamide gel with a 4% stacking gel casted over a 12.5% resolving gel. Bands were stained with Coomassie Brilliant Blue, excised from the gel, and protein enzymatic cleavage was carried out with trypsin (Promega; 1:20, *w*/*w*, Alexandria, NSW, Australia) at 37 °C for 16 h, as previously described [41]. Purification and concentration of peptides was performed using C18 Zip Tip Solid Phase Extraction (Millipore, Burlington, MA, USA).

Dried peptide samples were reconstituted with 2% ACN-0.1% FA (Acetonitrile-Formic acid), spiked with internal retention time peptide standards (iRT, Biognosys, Schlieren, Switzerland), and quantified with a NanoDropTM spectrophotometer (Thermo Fisher Sci., Waltham, MA, USA) prior to LC-MS/MS analysis using an EVOSEP ONE system coupled to an Exploris 480 mass spectrometer (Thermo Fisher Sci.). Peptides were resolved using C18 Performance column (75 µm × 15 cm, 1.9 µm particles; Evosep, Odense, Denmark) with a predefined Xcalibur Whisper100 20 SPD (58 min, IonOpticks, Aurora Elite, EV1112, Collingwood, VIC, Australia) method. Peptides were ionized using 1.6 kV spray voltage at a capillary temperature of 275 °C. Sample data were acquired in data-independent acquisition (DIA) mode with full MS scans (scan range: 400 to 900 *m*/*z*; resolution: 60,000; maximum injection time: 22 ms; normalized AGC target: 300%) and 24 periodical MS/MS segments applying 20 Th isolation windows (0.5 Th overlap: Resolution: 15,000; maximum injection time: 22 ms; normalized AGC target: 100%). Peptides were fragmented using a normalized HCD collision energy of 30%.

Mass spectrometry data files were analyzed using Spectronaut (Biognosys, Schlieren, Switzerland) by direct DIA analysis (dDIA). MS/MS spectra were searched against the Uniprot proteome reference from ETEC *E. coli* H10407 plus *S. flexneri* database using standard settings. Carbamidomethyl (C) was set as a fixed modification, and oxidation (M), acetyl (protein N-term), deamidation (N), and Gln-> pyro-Glu as variable modifications for total protein analysis.

Proteins quantified with at least two unique peptides, a *p*-value lower than 0.05, and an absolute fold change of <1 or >1 in linear scale were considered significantly differentially expressed.

### 4.7. Analysis of LTB Protein Presence

To verify the expression of the *eltb* gene and consequent presence of the LTB product in different fractions (bacteria and supernatants), a GM1 capture-ELISA was performed. It was carried out on a 96-well plate (Maxisorb; Nunc, Wiesbaden, Germany), coated with 100 μL of ganglioside GM1 (Sigma-Aldrich, Misuri, MO, USA) at 0.5 µg/mL in PBS at room temperature overnight. The plates were subsequently washed four times with PBS-Tween 0.05%. Non-specific binding sites were blocked with 5% milk in PBS for 1 h at room temperature. After washing, the different samples diluted in PBS were added, and the microplates were incubated at 37 °C for 2 h. The protein bound to the receptor was detected using a rabbit anti-LT (A + B) antibody (ab188541, Abcam, Cambridge, UK) at 1:8000 in PBS for 4 h, followed by a secondary anti-rabbit IgG conjugated with alkaline phosphatase (1:1000 in PBS, Nordic-MUbio, Susteren, The Netherlands). Finally, plates were revealed with an H_2_O_2_/4-Chloro-1-naphthol solution and absorbance was measured at 405 nm (Multiskan EX microplate photometer). Cholera toxin (0.1 µg/mL) was used as positive control (Sigma, St. Louis, Misuri, MO, USA).

### 4.8. Analysis of Biofilm Formation and LTB Implications

To evaluate the in vitro biofilm-formation capacity of the recombinants *S. flexneri::eltb* and *S. flexneri ΔtolR::eltb*, a previously established biofilm quantification method was used [42]. The strains (*S. flexneri*,* S. flexneri ΔtolR*, *S. flexneri::eltb*, *S. flexneri ΔtolR::eltb*) were incubated on TSA plates at 37 °C for 24 h. Colonies were collected to inoculate TSB and adjusted to O.D._600nm_ 0.125, from which 100 μL were used to inoculate 900 μL of TSB in 24-well plates (Corning^®^) and incubated at 37 °C for 2 days, changing the medium at 48 h. After this period, the wells were washed three times with PBS and dried at 60 °C for 1 h. Then, 1 mL of crystal violet was added to each well and incubated for 15 min at room temperature. After washing the wells five times with PBS, the crystal violet associated with the biofilms was extracted with 1 mL of alcohol–acetone (8:2). Quantification was performed by measuring absorbance at 550 nm in a spectrophotometer (Multiskan EX microplate photometer).

Subsequently, to evaluate the influence of the LTB protein on biofilm formation, the experiment was repeated with *S. flexneri 2a* and *S. flexneri::eltb* strains by adding 50 μL of GM1 at a concentration of 0.5 µg/mL in PBS to the wells. The plates were incubated for 4 days to promote a higher rate of biofilm formation. The rest of the procedure was the same as above.

### 4.9. Viability of Treated HeLa Cells

HeLa cells were maintained at subconfluence in 95% air and 5% CO_2_ humidified atmosphere at 37 °C. Complete RPMI medium supplemented with L-Glutamine (Gibco), 10% of fetal bovine serum (Gibco), and penicillin (10,000 units/mL)–streptomycin (10,000 g/mL) solution (Gibco) was used for maintenance. The MTT assay was used to measure cytotoxicity induced by the bacterial vesicles. Briefly, Hela cells (10^4^ cells/well) were incubated in 96-well flat bottom microplates (Falcon) for 24 h in complete RPMI medium. Cells were then treated with different concentrations (3.13–100 μg/mL) of OMVs or HT-OMVs *from S. flexneri 2a*, *S. flexneri ΔtolR*, *S. flexneri::eltb*, *S. flexneri ΔtolR::eltb* and incubated for 2 or 24 h. A mixture of 25 μL of MTT solution and 225 μL of RPMI medium was added and following 4 h of incubation, supernatants were removed, and 200 μL of pure DMSO (Panreac, Barcelona, Spain) was added to samples. Absorbance was measured at 540 nm (Thermo Scientific, Waltham, MA, USA).

### 4.10. Statistics

Statistical analyses were performed using GraphPad Prism9^®^ software (San Diego, CA, USA). All statistical significance analyses were carried out using the parametric one-way ANOVA test or the non-parametric Mann–Whitney U test as required. *p* values of <0.05 were considered to be statistically significant.

## 5. Conclusions

Here we present two novel *Shigella* strains, inserting a constitutive *eltb* gene from ETEC, as vectors to obtain OMV antigenic complexes containing both Shigella and ETEC antigens with the potential capacity to protect against both pathogens. Previous vaccine candidates targeting *Shigella* and *ETEC* have provided valuable insights into the development of protective strategies, such as live-attenuated vaccines like WRSS1 [43] for *Shigella* and the use of adjuvants like dmLT in *ETEC* vaccines. However, many of these candidates face challenges, including limited serotype coverage, the need for co-administration with adjuvants, and issues with stability and scalability. The OMV-based vaccine platform, incorporating LTB from ETEC, offers a novel approach that may overcome these limitations by providing broader protection and enhanced immunogenicity, while also ensuring ease of production and stability in resource-limited settings [44]. The newly engineered OMV vaccine candidates are not cytotoxic and express the main immunomodulatory and virulence factors of *Shigella*, with a similar expression level of LTB found in ETEC. This work shows for the first time the effectiveness of the genomic recombination of *eltb* in *Shigella*, obtaining a constitutive expression of the subunit nontoxic LTB as a potential OMV-based multiepitope vaccine platform against ETEC and *S. flexneri,* but also against other *Shigella* species like *S. sonnei* [23] in line with WHO recommendations. Further immunogenicity studies in animal models will be required for validation of the vaccine candidate.

## Figures and Tables

**Table 1:** Size distribution and zeta potential of the different OMV products.

Outer Membrane Vesicles	Mean Size (nm) [Polydispersity Index]	Zeta Potential (mV)
**OMV *S. flexneri***	193 [0.41]	−7.47
**OMV *S. flexneri::eltb***	271 [0.46]	−15.00
		
**HT-OMV *S. flexneri***	145 [0.20]	−12.18
**HT-OMV *S. flexneri::eltb***	165 [0.31]	−15.73
		
**OMV *S. flexneri ΔtolR***	403 [0.65]	−9.98
**OMV *S. flexneri ΔtolR::eltb***	200 [0.36]	−17.65
		
**HT-OMV *S. flexneri ΔtolR***	247 [0.29]	−15.83
**HT-OMV *S. flexneri ΔtolR::eltb***	157 [0.20]	−21.30

## Data Availability

Data is contained within the article.

## References

[1] Diarrhoeal Disease. https://www.who.int/news-room/fact-sheets/detail/diarrhoeal-disease.

[2] Diarrheal Diseases—Our World in Data. https://ourworldindata.org/diarrheal-diseases.

[3] Product & Delivery Research. https://www.who.int/teams/immunization-vaccines-and-biologicals/product-and-delivery-research/ppcs.

[4] WHO (2024). WHO Bacterial Priority Pathogens List. Bacterial Pathogens of Public Health Importance to Guide Research, Development and Strategies to Prevent and Control Antimicrobial Resistance.

[5] Olson S., Hall A., Riddle M.S., Porter C.K. (2019). Travelers’ Diarrhea: Update on the Incidence, Etiology and Risk in Military and Similar Populations—1990–2005 versus 2005–2015, Does a Decade Make a Difference?. Trop. Dis. Travel Med. Vaccines.

[6] Mirhoseini A., Amani J., Nazarian S. (2018). Review on Pathogenicity Mechanism of Enterotoxigenic *Escherichia coli* and Vaccines against It. Microb. Pathog..

[7] Bryson A., Gonzalez G., Al-Atoom N., Nashar N., Smith J.R., Nashar T. (2023). Extracellular Vesicles Are Conduits for *E. Coli* Heat-Labile Enterotoxin (LT) and the B-Subunits of LT and Cholera Toxin in Immune Cell-to-Cell Communication. Microb. Pathog..

[8] Zhang Y., Tan P., Zhao Y., Ma X. (2022). Enterotoxigenic *Escherichia coli* : Intestinal Pathogenesis Mechanisms and Colonization Resistance by Gut Microbiota. Gut Microbes.

[9] Berzosa M., Nemeskalova A., Zúñiga-Ripa A., Salvador-Bescós M., Larrañeta E., Donnelly R.F., Gamazo C., Irache J.M. (2022). Immune Response after Skin Delivery of a Recombinant Heat-Labile Enterotoxin B Subunit of Enterotoxigenic *Escherichia coli* in Mice. Pharmaceutics.

[10] Zhang W., Sack D.A. (2015). Current Progress in Developing Subunit Vaccines against Enterotoxigenic *Escherichia coli*-Associated Diarrhea. Clin. Vaccine Immunol..

[11] Nisa I., Qasim M., Yasin N., Ullah R., Ali A. (2020). Shigella Flexneri: An Emerging Pathogen. Folia Microbiol..

[12] Duncan-Lowey J.K., Wiscovitch A.L., Wood T.E., Goldberg M.B., Russo B.C. (2020). Shigella Flexneri Disruption of Cellular Tension Promotes Intercellular Spread. Cell Rep..

[13] Ranganathan S., Doucet M., Grassel C.L., Delaine-Elias B., Zachos N.C., Barry E.M. (2019). Evaluating Shigella Flexneri Pathogenesis in the Human Enteroid Model. Infect. Immun..

[14] Camacho A.I., de Souza J., Sánchez-Gómez S., Pardo-Ros M., Irache J.M., Gamazo C. (2011). Mucosal Immunization with Shigella Flexneri Outer Membrane Vesicles Induced Protection in Mice. Vaccine.

[15] Pastor Y., Camacho A., Gil A.G., Ramos R., de Ceráin A.L., Peñuelas I., Irache J.M., Gamazo C. (2017). Effective Protection of Mice against Shigella Flexneri with a New Self-Adjuvant Multicomponent Vaccine. J. Med. Microbiol..

[16] Pastor Y., Camacho A.I., Zúñiga-Ripa A., Merchán A., Rosas P., Irache J.M., Gamazo C. (2018). Towards a Subunit Vaccine from a Shigella Flexneri ΔtolR Mutant. Vaccine.

[17] Cassels F.J., Khalil I., Bourgeois A.L., Walker R.I. (2024). Special Issue on Enterotoxigenic *Escherichia coli* (ETEC) Vaccines: ETEC Infection and Vaccine-Mediated Immunity. Microorganisms.

[18] WHO Preferred Product Characteristics for Vaccines Against Enterotoxigenic Escherichia coli. https://www.who.int/publications/i/item/who-preferred-product-characteristics-for-vaccines-against-enterotoxigenic-escherichia-coli.

[19] WHO (2021). Preferred Product Characteristics for Vaccines Against Shigella.

[20] Walker R., Kaminski R.W., Porter C., Choy R.K.M., White J.A., Fleckenstein J.M., Cassels F., Bourgeois L. (2021). Vaccines for Protecting Infants from Bacterial Causes of Diarrheal Disease. Microorganisms.

[21] Medeiros P.H.Q.S., Bolick D.T., Ledwaba S.E., Kolling G.L., Costa D.V.S., Oriá R.B., Lima A.Â.M., Barry E.M., Guerrant R.L. (2020). A Bivalent Vaccine Confers Immunogenicity and Protection against Shigella Flexneri and Enterotoxigenic *Escherichia coli* Infections in Mice. NPJ Vaccines.

[22] Harutyunyan S., Neuhauser I., Mayer A., Aichinger M., Szijártó V., Nagy G., Nagy E., Girardi P., Malinoski F.J., Henics T. (2020). Characterization of ShigETEC, a Novel Live Attenuated Combined Vaccine against Shigellae and ETEC. Vaccines.

[23] Pastor Y., Calvo A., Salvador-Erro J., Gamazo C. (2023). Refining Immunogenicity through Intradermal Delivery of Outer Membrane Vesicles against Shigella Flexneri in Mice. Int. J. Mol. Sci..

[24] Schuch R., Maurelli A.T. (1997). Virulence Plasmid Instability in Shigella Flexneri 2a Is Induced by Virulence Gene Expression. Infect. Immun..

[25] Chernyatina A.A., Low H.H. (2019). Core Architecture of a Bacterial Type II Secretion System. Nat. Commun..

[26] Gabor C.E., Hazen T.H., Delaine-Elias B.C., Rasko D.A., Barry E.M. (2023). Genomic, Transcriptomic, and Phenotypic Differences among Archetype *Shigella flexneri* Strains of Serotypes 2a, 3a, and 6. mSphere.

[27] Berzosa M., Delgado-López A., Irache J.M., Gamazo C. (2023). Optimization of Enterotoxigenic *Escherichia coli* (ETEC) Outer Membrane Vesicles Production and Isolation Method for Vaccination Purposes. Microorganisms.

[28] Pakbin B., Brück W.M., Brück T.B. (2023). Molecular Mechanisms of Shigella Pathogenesis; Recent Advances. Int. J. Mol. Sci..

[29] Yagnik B., Sharma D., Padh H., Desai P. (2019). Oral Immunization with LacVax® OmpA Induces Protective Immune Response against Shigella Flexneri 2a ATCC 12022 in a Murine Model. Vaccine.

[30] Xu D., Zhang W., Zhang B., Liao C., Shao Y. (2016). Characterization of a Biofilm-Forming *Shigella Flexneri* Phenotype Due to Deficiency in Hep Biosynthesis. PeerJ.

[31] Fekete P.Z., Mateo K.S., Zhang W., Moxley R.A., Kaushik R.S., Francis D.H. (2013). Both Enzymatic and Non-Enzymatic Properties of Heat-Labile Enterotoxin Are Responsible for LT-Enhanced Adherence of Enterotoxigenic *Escherichia coli* to Porcine IPEC-J2 Cells. Vet. Microbiol..

[32] Berberov E.M., Zhou Y., Francis D.H., Scott M.A., Kachman S.D., Moxley R.A. (2004). Relative Importance of Heat-Labile Enterotoxin in the Causation of Severe Diarrheal Disease in the Gnotobiotic Piglet Model by a Strain of Enterotoxigenic *Escherichia coli* That Produces Multiple Enterotoxins. Infect. Immun..

[33] Mudrak B., Kuehn M.J. (2010). Heat-Labile Enterotoxin: Beyond G M1 Binding. Toxins.

[34] Horstman A.L., Kuehn M.J. (2002). Bacterial Surface Association of Heat-Labile Enterotoxin through Lipopolysaccharide after Secretion via the General Secretory Pathway. J. Biol. Chem..

[35] Santiago-Mateo K., Zhao M., Lin J., Zhang W., Francis D.H. (2012). Avirulent K88 (F4)+ *Escherichia coli* Strains Constructed to Express Modified Enterotoxins Protect Young Piglets from Challenge with a Virulent Enterotoxigenic *Escherichia coli* Strain That Expresses the Same Adhesion and Enterotoxins. Vet. Microbiol..

[36] Köseoğlu V.K., Hall C.P., Rodríguez-López E.M., Agaisse H. (2019). The Autotransporter IcsA Promotes Shigella Flexneri Biofilm Formation in the Presence of Bile Salts. Infect. Immun..

[37] Akhtar M., Basher S.R., Nizam N.N., Hossain L., Bhuiyan T.R., Qadri F., Lundgren A. (2023). T Helper Cell Responses in Adult Diarrheal Patients Following Natural Infection with Enterotoxigenic *Escherichia coli* Are Primarily of the Th17 Type. Front Immunol.

[38] Llobet E., March C., Giménez P., Bengoechea J.A. (2009). *Klebsiella pneumoniae* OmpA Confers Resistance to Antimicrobial Peptides. Antimicrob. Agents Chemother..

[39] Camacho A.I., Souza-Rebouças J., Irache J.M., Gamazo C. (2013). Towards a Non-Living Vaccine against Shigella Flexneri: From the Inactivation Procedure to Protection Studies. Methods.

[40] Zúñiga-Ripa A., Barbier T., Lázaro-Antón L., de Miguel M.J., Conde-Álvarez R., Muñoz P.M., Letesson J.J., Iriarte M., Moriyón I. (2018). The Fast-Growing Brucella Suis Biovar 5 Depends on Phosphoenolpyruvate Carboxykinase and Pyruvate Phosphate Dikinase but Not on Fbp and GlpX Fructose-1,6-Bisphosphatases or Isocitrate Lyase for Full Virulence in Laboratory Models. Front. Microbiol..

[41] Shevchenko A., Tomas H., Havli J., Olsen J.V., Mann M. (2006). In-Gel Digestion for Mass Spectrometric Characterization of Proteins and Proteomes. Nat. Protoc..

[42] O’Toole G.A. (2011). Microtiter Dish Biofilm Formation Assay. J. Vis. Exp..

[43] Hartman A.B., Venkatesan M.M. (1998). Construction of a Stable Attenuated *Shigella Sonnei* Δ *VirG* Vaccine Strain, WRSS1, and Protective Efficacy and Immunogenicity in the Guinea Pig Keratoconjunctivitis Model. Infect. Immun..

[44] Yin J., Wu H., Li W., Wang Y., Li Y., Mo X., Li S., Ren Y., Pan H., Jiang P. (2024). *Escherichia coli* Heat-Labile Enterotoxin B Subunit as an Adjuvant of Mucosal Immune Combined with GCRV-II VP6 Triggers Innate Immunity and Enhances Adaptive Immune Responses Following Oral Vaccination of Grass Carp (*Ctenopharyngodon idella*). Fish Shellfish Immunol..

